# Biosurfactants: Forthcomings and Regulatory Affairs in Food-Based Industries

**DOI:** 10.3390/molecules28062823

**Published:** 2023-03-21

**Authors:** Deepansh Sharma, Deepti Singh, Gadhwal Monika Sukhbir-Singh, Bhoomika M. Karamchandani, Gajender Kumar Aseri, Ibrahim M. Banat, Surekha K. Satpute

**Affiliations:** 1 Department of Life Sciences, J. C Bose University of Science & Technology, YMCA Faridabad-Haryana, Haryana 121006, India; 2 Amity Institute of Microbial Technology, Amity University Rajasthan, Jaipur 303002, India; 3 Department of Microbiology, Savitribai Phule Pune University, Pune 411007, India; 4 School of Biomedical Sciences, Faculty of Life and Health Sciences, University of Ulster, Coleraine BT52 1SA, UK

**Keywords:** antimicrobial, acute toxicity, biosurfactants, food additives, generally regarded as safe (GRAS) strains

## Abstract

The terms discussed in this review—biosurfactants (BSs) and bioemulsifiers (BEs)—describe surface-active molecules of microbial origin which are popular chemical entities for many industries, including food. BSs are generally low-molecular-weight compounds with the ability to reduce surface tension noticeably, whereas BEs are high-molecular-weight molecules with efficient emulsifying abilities. Some other biomolecules, such as lecithin and egg yolk, are useful as natural BEs in food products. The high toxicity and severe ecological impact of many chemical-based surfactants have directed interest towards BSs/BEs. Interest in food surfactant formulations and consumer anticipation of “green label” additives over synthetic or chemical-based surfactants have been steadily increasing. BSs have an undeniable prospective for replacing chemical surfactants with vast significance to food formulations. However, the commercialization of BSs/BEs production has often been limited by several challenges, such as the optimization of fermentation parameters, high downstream costs, and low yields, which had an immense impact on their broader adoptions in different industries, including food. The foremost restriction regarding the access of BSs/BEs is not their lack of cost-effective industrial production methods, but a reluctance regarding their potential safety, as well as the probable microbial hazards that may be associated with them. Most research on BSs/BEs in food production has been restricted to demonstrations and lacks a comprehensive assessment of safety and risk analysis, which has limited their adoption for varied food-related applications. Furthermore, regulatory agencies require extensive exploration and analysis to secure endorsements for the inclusion of BSs/BEs as potential food additives. This review emphasizes the promising properties of BSs/BEs, trailed by an overview of their current use in food formulations, as well as risk and toxicity assessment. Finally, we assess their potential challenges and upcoming future in substituting chemical-based surfactants.

## 1. Introduction

The term ‘food additives’ represents the substances that are added to food to retain or preserve and/or improve some physical properties, often taste, texture, freshness and appearance, along with its safety. However, it is additionally imperative to evaluate the food additives themselves for their potential harmful effects on human health before they are utilized for their desired applications. Among several substances available, surfactants are considered as the most multifarious agents explored for varied applications, such as detergents, pesticide application, cosmetics and microbial enhanced oil recovery processes, and the food-processing industry [1,2,3]. Surfactants are obtained from various sources, e.g., petrochemicals, fatty acids, microbial cells, etc. [4,5]. Some known natural surfactants, such as lecithin from egg yolk and milk proteins, are prominently used in salad dressings and for the enhancement of flavor, appearance, and texture of desserts [6,7]. The growing interest in surfactants and the identification of appropriate molecules with less toxicity and efficient surface characteristics have been of immense interest for both industrial and scientific communities.

Synthetic surfactants are linked with many health-related issues and drawbacks, among which intestinal dysfunction [8] is reported prominently. Surfactants are used in foods in relatively high concentrations, which might lead to severe intestinal permeability, which in turn may elicit various allergic and autoimmune diseases [9]. Surfactants increase intestinal permeability for a limited time in a precise and recurrent way in the presence of antigens and pathogens. It is crucial to note that there are no acceptable daily intake (ADI) guidelines for the use of surfactants in food production [10]. The ADI guidelines specify the highest amount or the limit of a particular chemical which can be consumed regularly over the period of a life span without any health-related issues or apparent side effects. Demands of green ingredients over synthetic additives (“green label”) have led to extensive research in pursuit of new microbial sources for the production of effective surface-active or emulsifying agents [11,12]. 

BSs are generally low-molecular-weight compounds with the ability to reduce surface tension noticeably, whereas BEs are high-molecular-weight molecules with efficient emulsifying abilities. BSs represent surface-active compounds of microbial origin. BEs are considered as BSs that are used as emulsifiers; therefore, the term BS is much wider and inclusive of BEs. Multifarious BS/BE molecules confer or provide various functional properties to food, such as emulsifying, additive, foam-forming, and wetting agents, in addition to pharmaceutical-related properties (antimicrobial, antiadhesive, antiviral, antibiofilm, etc.) [12,13]. Even though BSs and BEs have an unquestionable potential for replacing synthetic surfactants, with huge importance to food industries. Major blockages include high production cost and apprehension regarding their safety. The present research about BSs/BEs in food production is restricted to laboratory conditions, without detailing any assessment regarding their safety and hazard analysis, which has restricted their acceptance for several food-related applications [12,13,14]. It is important to note that committed guidelines for adopting BSs/BEs in food formulations do not exist; however, the recommendations to include them as all-purpose food additives might be accepted, and would grant their primary approval. In the present review, we comprehensively discuss the roles of BSs/BEs as food additives for quality and texture improvement, along with challenges, and forthcoming regulatory considerations. 

## 2. Biosurfactants as Food Additives 

Food additives are compounds or substances that facilitate the enhancement of overall food properties. Since ancient times, some food additives such as salt, sugar, and SO_2_, have been utilized to preserve meats, fishes, beverages, etc. Currently, the journey of food additives has made huge advances, from kitchens and small factories to the advanced commercial scale. Food additives have been obtained from various natural sources, or synthesized chemically, and added to food items to achieve some positive technological benefits. Thus, food additives should solely serve the intended desired aims of preserving the nutritional quality of food, and not result in any negative effects. Based upon their functional properties, the World Health Organization (WHO) and the Food and Agriculture Organization (FAO) have broadly categorized additive compounds as (1) flavoring agents, (2) enzyme preparations, and (3) other additives. 

Microbial surfactants as food additives comprise molecules that may be introduced to food in order to confer emulsifying, foaming, thickening, texture-improving, and/or preserving properties, along with the encapsulation of fat-soluble substances such as vitamins (called “direct food additives”). Attributes such as antiadhesive/antimicrobial or food surface cleaning are termed as “indirect food additives” that are fulfilled through packaging, coating, or transport and storage processes. Microbial BSs pose antiadhesive and antimicrobial activity against several pathogens and have been listed in Table 1. BSs may interact with porins (proteins of cross cellular membranes) and may lead to leakage of the cytoplasmic content of the cell, resulting in cell death [13]. However, prior to the inclusion of BSs/BEs in food processing, they must undergo critical toxicological assessment protocols which test synergistic compatibility with food molecules, dosage limit determinations for daily intake, and potential protective effects of their use. Rhamnolipids (RLs) have been reported to improve various properties, such as dough stability, batter texture, and the volume and shape of bakery products [14]. A patent on ‘RLs in bakery products’ emphasizes the improvement of dough characteristics and the volume of bakery products after mixing with RLs [15]. Basically, L-rhamnose is a methyl pentose natural sugar found in varied microbial RLs [16], which is useful as a food additive.

**Figure 1:**
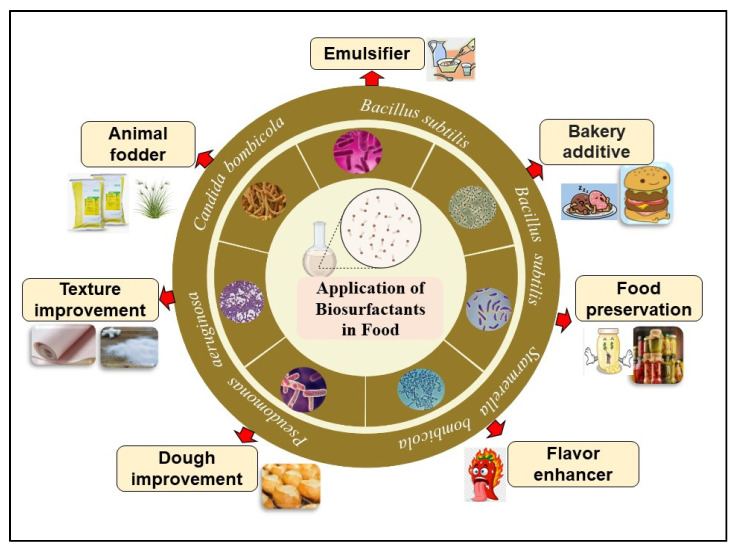
Different roles of BSs/BEs in improving properties of food, including taste, texture, and flavor improvements.

Kiran et al. [17] described the BSs of *Nesterenkonia* sp. for the enhancement of muffin texture. The *Nesterenkonia* sp. are obligate aerobes, grouped under the genus *Micrococci,* which grow optimally between 25 and 37 °C. Phylogenetic and chemotaxonomic analysis of isolates showed *Kocuria*, *Kytococcus Dermacoccus,* and *Nesterenkonia* under the genus *Micrococcus* [18]. Use of *Nesterenkonia* provides various additional benefits to the muffins, including a decrease in hardness, chewiness and gumminess compared to control treatments in the presence of 0.75% lipopeptide in the preparation mixture. The roles of microbial BSs/BEs (e.g., surfactin, RLs, lipopeptides, glycolipids, and emulsan) as emulsifiers, bakery additives, flavor enhancers, bread improvers, etc., are outlined in Figure 1 and listed in Table 2.

**Table 1:** Antiadhesive and antimicrobial roles of various microbial-originated biosurfactants against pathogens.

**ANTIADHESIVE**			
Microorganisms	Biosurfactant	Pathogens	Reference
*Pseudomonas putida*	Putisolvin I and II	*Pseudomonas* sp.	[19]
	Psedofactin II	*Enterobacter faecalis* *Proteus mirabilis, Candida* sp.	[20]
*Bacillus subtilis*	Fengycin	*Salmonella enterica*	[21]
*Bacillus tequilensis*	Lipopeptide	*Streptococcus mutans*	[22]
*Candida spaerica*	Lunasan	*Streptococcus agalactiae * *Pseudomonas aeruginosa*	[23]
*Pseudomonas aeruginosa*	Rhamnolipid	*Yarrowia* sp.	[24]
*Candida lipolytica*	Rufisan	*Streptococcus* sp.	[25]
*Serretia marsecens*	Glycolipid	*Candida albicans* *Pseudomonas aeruginosa* *Bacillus pumilus*	[26]
**ANTIMICROBIAL**			
Microorganisms	Biosurfactant (MIC µg/mL)	Pathogens	Reference
*Pseudomonas aeruginosa*	Rhamnolipids (4–64)	*Alternaria alternata* *Aureobasidium pullulans* *Aspergillus niger* *Candida albicans* *Chaetonium globosum* *Gliocadium virens*	[27]
*Pseudomonas aeruginosa*	Rhamnolipids (20–50)	*Alternaria mali* *Brotrytis cinerea* *Fusarium* sp. *Rhizoctonia solani*	[28]
*Pseudomonas aeruginosa*	Rhamnolipids (0.5–1.70)	*Brotrytis cinerea* *Fusarium* sp. *Fusarium solani* *Gliocadium virens* *Penicillium funiculosum* *Rhizoctonia solani*	[29]
*Pseudomonas aeruginosa*	Rhamnolipids	*Brotrytis cinereal*	[30]
*Pseudomonas aeruginosa*	Rhamnolipids (64–256)	*Brotrytis cinereal* *Mucor miehei * *Staphylococcus aureus* *Bacillus cereus*	[31]
*Pseudomonas* sp.	Rhamnolipid	*Pseudomonas aeruginosa*	[32]

**Table 2:** Potential applications of biosurfactant bio emulsifiers in food system.

**FOOD ADDITIVES**			
Microorganisms	Biosurfactant	Applications	Reference
*B. subtilis*	Surfactants	Emulsifier	[33]
*C. utilis*	-	Mayonnaise emulsifier	[34]
*B. subtilis*	-	Bakery additive	[35]
*B. subtilis*	Surfactin	Food preservative	[36]
*Pseudomonas sp.*	Rhamnolipid	Dough improvement	[15]
*Pseudomonas aeruginosa*	Rhamnolipid		[16]
*Nesterenkonia sp.*	Lipopeptides	Texture improvement	[17]
*Bacillus subtilis*	-	Cookie dough	[37]
*Bacillus subtilis*	Lipopeptides	Bread improvement	[38]
*Candida bombicola*	Glycolipids	Cupcake additive	[39]
*Starmerella bombicola*	Sophorolipids	Sophorolipids + curcumin	[40]
*Probiotic* (GRAS)	-	Animal fodder	[41]
**EMULSIFICATION**			
Microorganisms	Biosurfactant type	Emulsification material	Reference
*Bacillus vallismortis*	Exopolysaccharides	Essential oils	[42]
*Pseudomonas fluorescens*	Exopolysaccharides	Edible oils	[43]
*Nesterenkonia* sp.	Lipopeptide	Unsaturated hydrocarbons	[18]
*Candida utilis*	Glycolipids	Vegetable oil	[44]
*Pseudomonas aeruginosa*	Rhamnolipids	Saturated hydrocarbons	[45]
*Kluyveromyces marxianus*	Mannoprotien	Corn oil	[46]
*Saccharomyces lipolytica*	-	Cooking vegetable oil	[47]
*Candida utilis*	Glycolipids	Canola oil	[48]
*Pseudomonas aeruginosa*	Rhamnolipids	Nano-emulsion	[49]

Campos et al. [34] established the varied formulations of mayonnaise with *Candida utilis* derived BE as a key ingredient to confer stability to the emulsion during storage process. Dough properties and volume were considerably improved with the use of the chemical emulsifier glycerol monostearate at the application of 0.1% BSs. In another example *B. subtilis*-derived surfactants were reported highlighting their ability to enhance dough structural properties and the textural quality of cookies [37]. Similarly, Mnif et al. [38] reported the enhancement of bread dough quality with a *B. subtilis*-derived BS at a concentration of 0.075% (*w*/*w*) in comparison to soya lecithin. Other additional improvements in the structural properties of bread, such as chewiness, cohesion, and reduction in firmness were also reported. 

In a bakery-related application, Silva et al. [39] had incorporated a BS into cupcakes as a replacement of 50–100% of the plant fat contents. The replacement of plant fat by a BS resulted in some improvement in the nutritional value of the cupcake, through the reduction of trans-fatty acids (prevalent in plant fat). Microbial BSs have also been explored to improve animal feed by enriching rapeseed meal with GRAS microorganisms. Much longer ago (1951), it was already well known that surfactants encourage the growth of chickens [50]. It was also later suggested that non-ionic surfactants do have an additional impact on animals, in improving their weight, milk production capacity, and feed hydrolysis [51]. Enriching rapeseed meal with BSs produced by GRAS microorganisms successfully hydrolyzes the rapeseed meal and provides several benefits in terms of probiotic concepts. Therefore, BSs can be used as a magnificent substitute to antibiotics, some of which are restricted for use in animal feed [41].

The emulsification activity of BS/BE molecules is decisive for food industries and can be predicted by thorough understanding of their hydrophilic-lipophilic balance (HLB), which designates their usage in the preparations of water-in-oil (W/O) or oil-in water (O/W) emulsions. Based on the HLB scale (0–20), each BS/BE can be categorized, in order to explore them further for suitable applications. HLB values between 3 and 6 are desired for W/O microemulsions, while those between 8 and 18 buoy up O/W microemulsions. For instance, RLs, surfactin, and sophorolipids (SLs), according to their HLB values, favor the improvement of O/W emulsions. Some of the HLB values for representative BSs and Polysorbate 80 (as a reference) are listed in Table 3 [52]. 

The foremost role of surface-active agents is dropping the interfacial tension that permits the formation of small droplets in an insoluble liquid (oil and water). Surfactants reduce adverse interactions between a water–oil (W/O) interface and permit the dispersion of droplets of one phase into the other. The decrease in the droplet size of an emulsion improves the stability of suspension or liquid solutions [53]. Another potential application of BSs and BEs is their ability to form micro-emulsions, which can be utilized as carriers for fat-soluble vitamins and value-added molecules [54]. Research published by Farheen et al. [55] suggested *P. aeruginosa*-derived RLs, based in nano-BS preparation and its application in bakery industry facilitating enhanced emulsifying potential, as equated to synthetic surfactants.

**Table 3:** HLB value evaluation of different surfactants.

Type of Biosurfactants	HLB Value	Reference
Rhamnolipids (RLs)	10.17	[56]
Sophorolipids (SLs)	10–13	[57]
Surfactin	10–12	[52]
Mannosylerythritol lipid (MEL)	≥12	[58]
Other glycolipids	10–15	[59]
Lipopeptides	10–11.1	[60]
Polysorbate 80	14.4–15.6	[61]

**Figure 2:**
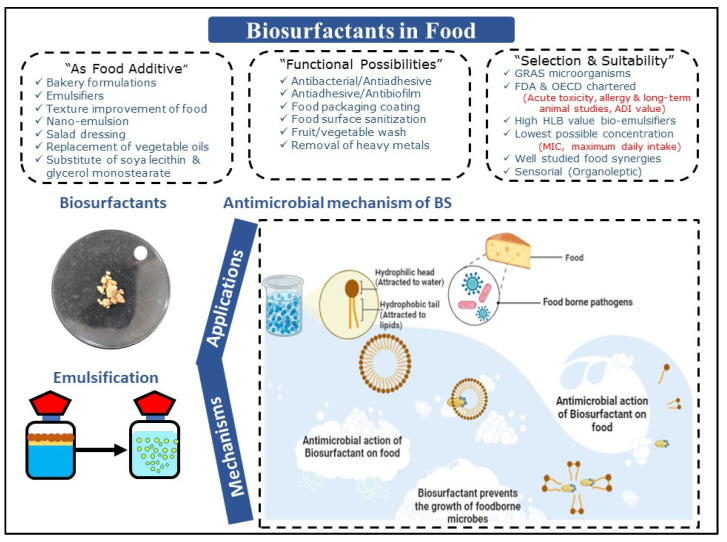
Applications of biosurfactants/bioemulsifiers in food, including their antimicrobial activities against pathogens.

SLs are recognized for their substantial emulsification potential towards vegetable oil utilized in bakery preparations. Gaur et al. [62] reported the production of SLs by *Candida* spp. and further explored its applications as an emulsifier for the food industry. BSs exhibited substantial emulsification activity with olive (51%), soybean (39%), almond (50%), and mustard (50%) oils. It is a well-established fact that BSs can act as efficient emulsifying agents for several oils, and thus can probably be used in several food-related applications. Various other “indirect” applications, including biocidal, food preservation, and antibiofilm applications, need more substantiation and standardization protocols, environmental aspect assessments, along with synergy-supporting evidence [36,37]. Overall, applications of BSs/BEs in food, including their antimicrobial activities against pathogens, are certainly promising and are represented in Figure 2.

## 3. Sensorial Behavior 

In addition to the physical-chemical properties, other indispensable traits, such as sensorial behavior pattern, stability, and shelf life to food products may also be contributed by BSs and BEs. Due to their exceptional antimicrobial, antibiofilm, and antiadhesive potentials, these BSs and BEs directly affect and/or impede the growth of pathogens in processed foods [63,64]. Ozdener et al. [65] extracted SLs from *Starmerella bombicola* ATCC 22214 and demonstrated the sensorial properties of this BS. In the literature, SLs have been reported frequently from nonpathogenic yeast species from the genus *Candida* by growing them on renewable or cheaper substrates. SLs are used generally for the preparation of formulations for the use of cleaning solutions, as well as laundry and/or dishwashing disinfectants. Because of the uniqueness in the chemical structure of SLs, researchers have checked these surface-active molecules for properties, such as those affecting taste. The sensory characteristics of SLs were evaluated by Ozdener et al. [65] using cultured human fungiform taste papillae (HBO) cells. SLs have oral taste sensory acceptance, which widens their possibilities for food formulations, in order to mitigate bitter sensory properties in foods and drugs. 

The broadened application potential of SLs has been demonstrated to capture the potential for nondetectable sweet dysgeusia in taste sensory sensation [65]. Sweet dysgeusia is associated with an unusual symptom which causes the individual to experience the perception of sweet taste for all kinds of food. The reason behind this distortion or condition has not been established yet. While its reason is still unidentified, it has been progressively described in the settings of lung cancer and syndrome of the unbalanced release of antidiuretic hormone-related hyponatremia (a condition in which sodium concentration in the blood is atypically low). The findings of such evaluations open up further potential for the practical applications of SLs for improving the bitter taste of foods and drugs, and improving drug-intake acquiescence by patients. 

The combined characteristics of SLs as BSs, antibiocidal agents, and sensory taste-stimulating substances will meaningfully increase the commercial value of such glycolipids. Detailed study in these proposed areas has opened new opportunities for applied aspects of BSs as masking and/or blocking substances which cause the bitter-taste perception of foods and drugs. Bitter-taste insight is an innate trait and induces aversive reactions for some food components. Blocking/reducing bitter-taste perception is a crucial parameter for the acquiescence and acceptability of some foods and medicines. Thus, SLs represent an ideal candidate for use in future drugs and other food product design, due to their decidedly specific positive interactions with T1R3 sweet taste receptors. 

## 4. Food Matrix Interfaces 

Even with the vast potential of BSs and BEs in food formulations, it would be misleading not to debate the efficiency of BSs without exploring their synergies with food particles. High-molecular-weight biosurfactants (HMWBSs) possess protein and polysaccharide moieties that are valuable for many food formulations. Head groups of these BSs bind to the charges present on proteins. Typically, HMWBSs are highly competent emulsifying agents. The anionic surfactant binding is synchronized with pH condition, along with supportive hydrophobic exchanges. Industrial use of BSs and BEs requires the following characteristics: (i) the lowest critical micelle concentration (CMC) - crucial to produce small droplets; (ii) production of the lowest droplet size; (iii) stability at a wide range of pH, ionic strengths, and high temperatures [65]. 

For achieving the antimicrobial prospective of BSs, their synergistic potential needs to be determined. Magalhães and Nitschke [66] reported synergistic bacteriostatic activity of BS when conjugated with nisin (polycyclic peptide) produced by *Lactococcus lactis*. Nisin has huge antibacterial potential and is used frequently as food preservative. Researchers reported synergism between BS and nisin which act on the cell membrane. The majority of fermented food (natto and plant-based fermentation) pathogens can be inhibited by bacteriocins producing *Lactobacilli* and BSs producing *B. subtilis* [67]. Attention towards the selection of appropriate matrix, low dose, and minimum daily intake quantities not only escalates their efficacy, but also contributes to overcoming the issues of their chemical equivalents. Accordingly, there is a general demand to determine the right combinations that would enhance the role of BSs in food production. The combination of BSs with bacteriocins and other orthodox food preservatives is beneficial for the development of active antimicrobial food packaging material and for conferring antimicrobial potential with synergistic effects. Furthermore, SLs with asymmetric structures are able to form self-assemblies with exclusive functionality. This concept has led to the assumption that glycolipids being amphiphilic in nature can span through the structurally similar cell membrane. This also facilitates the entry of low concentrations of antimicrobials in order to achieve the anticipated potential of resisting substance abuse in biological systems.

## 5. Regulations to Chartered BSs and BEs as Food Additives 

The adoption of BSs and BEs is vital in order for them to be established as food additives, as they essentially possess the functional characteristics to attain droplet coalescence and are nontoxic in nature [68,69]. No defined governing recommendations exist to date that charter any BSs or BEs as food additives. We must adhere to the regulatory framework and time required for taking a product or process from laboratory to the market for commercialization purposes. Acute toxicity (LD_50_ dose) and allergy assessments are also needed before a substance can be established as a food additive [69]. The importance of the major end points, as recommended by the Organization for Economic Co-operation and Development (OECD), governs the rules for the risk evaluation of food additives. All of the rules are based on acute toxicity, allergic responses, reproductive toxicity, and mutagenic behavior; thus, these can also be assumed for the safety evaluation of BSs and BEs.

Campos et al. [34] demonstrated the addition of glycolipids as a food additive. Researchers used 0.7% (*w*/*v*) glycolipids as a food additive in mayonnaise production. The intake of about 15 g of treated food by an adult weighing 50 kg would be equivalent to 0.10 g of BSs, which equates to 2 mg/kg of total body weight. The dose of 2 mg/kg is much lower than directed to determine acute toxicity in laboratory animals (3600 mg/kg), which led to assuming no acute risk [33]. The US Food and Drug Administration (FDA), the FAO, and the WHO recommended that ADI should be documented to deliver “an adequate edge of safety and reduce health hazards in all groups”. The ADI denotes the amount of a food additive expressed or established per unit of body weight that can be ingested daily over a lifetime with no appreciable health risk. ADI approximates the amount of a food additive, expressed on a body weight basis, that can be administered daily while lacking considerable health risk. ADIs are only assigned to those food additives that are considerably cleared or removed from the body within 24 h.

One of the main classes of glycolipid BSs is that of RLs, which are primarily produced by the opportunistic pathogenic strains *P. aeruginosa*. This understanding led to the active search for alternative RL-producing strains [70]. Tripathi et al. [71] reported an RL-like BS-producing strain belonging to the genus *Marinobacter*. This strain showed no pathogenicity when evaluated using the *Galleria mellonella* infection model. Such strains expand the paradigm of RL biosynthesis to a new non-genetically-engineered bacteria, which may have prospects for food-related applications due to their potential to be synthesized from cheap, renewable feed stocks. Nonpathogenic BS-producing organisms are momentous, with reduced pathogenicity as compared to the pathogenic *P. aeruginosa* strains. Commercial production of RLs as possible food additives has been in progress in the USA (Jeneil Biotech, Saukville, WI, USA) with no defined hazards [72]. Jeneil Biotech produces natural BS molecules which can be utilized as promising alternatives over synthetic agents for various purposes, including the preservation of fruits and vegetables [72]. 

Microbial BSs fermentation processes utilize several renewable substrates for making their production economically feasible [73]. Commercial production of RLs, SLs and MELs from various strains is also underway considerably worldwide [73]. The Organization for Economic Cooperation and Development (OECD) is a distinguished forum that comprises the governments of 37 democracies, who work dedicatedly to design standard policies (with market-based economies) that encourage livable economic growth. A German-based industry, ‘Evonik’, is on track for the commercial production of SLs. Evonik produces SLs that are compliant with the regulations of OECD 301 F, which is related to aerobic biodegradability, and ISO 11,734 (anaerobic biodegradability) with an index of 100% Renewable Carbon Index (RCI). The SLs from Evonik achieve encouragingly better products than chemical surfactants when assessed for water toxicity (OECD 211 and 202). SL production with improved yields has been achieved using genetically improved yeast with reduced production costs (US Patent in 2015). 

Food ingredients with additives permitted after endorsement from the United States Department of Agriculture (USDA) for explicit use as BSs after labeling or identified as “GRAS” can be employed for potential applications. BSs obtained from *C. utilis* have been listed as GRAS organisms in the Code of Federal Regulations (The United States), originating from the federal Department of Health and Human Services, USA Food and Drug Administration (FDA) regulations, Title 21 (21CFR-172.590). The list comprises approved food additives, based on a history of safety, which can be used in foods. Moreover, microorganism-initiated ingredients may be the subject of a GRAS notice (FDA, 2010). For example, various lactic acid bacteria could be used as food additives in order to govern frameworks and rationalize BSs, as they are all permitted GRAS strains. Eco-friendly, biodegradable, and safe materials need to be explored in detail for future applications [10,74,75].

Microbial surfactants are chemically composed of hydrophobic (lipids) and hydrophilic (sugars, proteins/peptides, and acids) moieties [75,76]. The moieties present in various known BSs are nontoxic in due their simple and biodegradable nature. However, the origin of BSs from soil-borne and opportunistic pathogens such as *P. aeruginosa* demands a thorough analysis of their toxicity. It can be observed that the reports explaining the cytotoxic assessment of BSs are limited, and considering their origin along with their unique functional attributes, it should be essential to assess the toxicity obtained from different strains [76]. Therefore, it should also be essential to determine the toxicity of the BSs/BEs to be considered for food formulations. Similar methods for the assessment of the cytotoxicity of the materials to be used in biomedical and human industries need to be established as per the European standards (UNE EN ISO 10993-5:2009) [77] and guidelines for the biological evaluation of medical devices for in vitro cytotoxicity. A similar approach has been adopted for the cytotoxicity assessment of BSs produced by *L. pentosus* [75]. It was established that BSs obtained from this strain did not display any cytotoxicity up to a 1 g/L concentration. Other studies on BSs produced by marine strains of *Marinobacter* and *Pseudomonas* also showed a lack of cytotoxicity in in vitro models of human skin and liver cell investigations [78,79]. In this sense, it can be easily established that low concentrations of BSs have no cytotoxicity on human health, and the same can be anticipated for the adoption of BSs in food formulations. BSs with high efficiency at low concentrations could also help to maintain a low ADI value. An indirectly low ADI means a minimal requirement to attain the functionality of the molecule in food production, which is actually effectual for minimizing the cost of BS adoption. 

## 6. Antimicrobial Activity of Biosurfactant

Around 1.3 billion tons of food are lost around the globe annually, which results in the malnourishment of around one in nine people. Such food losses have been reported in a variety of vegetables, fruits, oilseeds, and in the meat and dairy industries [80]. The main reason for this problem is the deterioration of food quality, spoilage due to microbial attack, and the short shelf life of some foods. At present, several strategies for dealing with those challenges through the application of bio-based packaging material or antimicrobial agents are under investigation [81]. The existing research suggests the implementation of BSs, chitosan, bacteriocins, essential oils, and bio-based packaging material as potential processes for tackling this problem [82,83,84]. Recently, Kourmentza et al. [85] reported the antimicrobial activity of a lipopeptide BS against foodborne pathogens, such as *Bacillus* sp. They also reported the antimicrobial action against filamentous fungi, namely *Candida krusei* (MIC = 16–64 mg/L), *Paecilomyces variotti,* and *Byssochlamys fulva* (MICs = 1–16 mg/L).

BSs are believed to induce disruption of plasma membranes, leading to a buildup of intramembranous elements in the microbial cells that results in enhancement of the electrical conductance of the cell membrane [86]. It is also known that BSs affect the fatty acid composition in the plasma membrane, which results in disruption of the membrane’s permeability. BSs have also been reported to directly interact with the membrane lipids and trigger inhibition of some confined enzymes [87,88]. The antimicrobial activity of any compound depends upon the food matrix [89]. Food composition varies in proteins, carbohydrates, lipids, natural enzymes, inhibitors, and various other chemical constituents. The food matrix has a crucial role in the synergy of the antimicrobial activity of BSs. It can therefore be hypothesized from the above discussion that the presence of various enzymes, such as proteases, lipases, and other hydrolyzing enzymes, may affect the functionality and structural integrity of the surfactants. The use of BS-based antimicrobial compounds in food, therefore, requires proper evaluation of the impact of the food matrix on the functionality of these BSs. Understanding such an important aspect would be essential prior to the use of such BSs in food products.

## 7. Challenges and Forthcomings

BSs are finding their way into an increasing range of commercial products; however, the specific surface-active agents currently available are extremely limited and, consequently, do not always have the suitable physical-chemical characteristics to satisfy the formulation requirements of many products. As a result, chemical surfactants, which take many different forms, cannot always be replaced with a biologically produced equivalent. The two major glycolipid BSs, SLs and RLs, exhibit the widest potential for commercialisation. For RLs in particular, although many different wild-type bacteria have been reported to produce them, none of the organisms that produce them have high enough yields to be commercially viable. Therefore, as with many BSs, they fail to reach the marketplace due to two factors: yield and cost of production. 

Despite extensive possible applications, microbial BSs and BEs are not yet employed as additives at a substantial scale commercially. Furthermore, regulations governing the usage of new ingredients necessitate considerable research and safety assessment in order to charter approvals for food inclusion. However, commercialization is often inadequate due to challenges in process design, downstream processing, and low yields. All of the aforementioned factors influence the monetary input for the BS and BE industries, as compared with chemical surfactants. Nevertheless, BSs have an indisputable potential for substituting conventional surfactants, with prodigious advantages to food industries. 

Employing appropriate approaches in exploring applications of BSs and BEs in food industries is imperative. Researchers need to channel their findings in unique ways toward BSs with high HLB values, which would be beneficial for applications related with emulsification processes. BSs possessing antimicrobial potential with low MIC values should be used for preservative purposes. To rule out the toxicity issue, we must select strains with nonpathogenic or GRAS status for BS and BE production. The BSs and BEs obtained from GRAS strains may be the future choice due to their compliance with FDA guidelines (Food additive amendment, 1958), i.e., “GRAS ingredients”. There should, therefore, be a focus on the availability of a wider range of BS congeners for varying and potentially new-to-market commercial applications produced using nonpathogenic bacterial species.

An example of “slippery surfactants” (a probiotic-based product, i.e., PreLiminate^®^) used to accomplish surface colonization with advantageous bacteria appears to be helpful in eliminating biofilms in the food industry. The resistance evolved by food pathogens to microbial surfactants is also an unknown field, which needs to be considered for their active use as antimicrobial agents. In addition, the sensorial impact of BSs/BEs, in addition to their interaction with food components, needs to be further investigated. An innovative trend is to produce a tailored enzymatic synthesis of BSs, Glysosurf^®^, which appears to be promising. The unique properties presented by microbial BSs/BEs propose the future of molecules to be utilized the food processing chain, whether as additives, surface modifiers, and/or cleaning agents.

## Data Availability

Not applicable.

## References

[1] Sharma D., Saharan B.S. (2016). Functional characterization of biomedical potential of biosurfactant produced by *Lactobacillus helveticus*. Biotechnol. Rep..

[2] Satpute S.K., Kulkarni G.R., Banpurkar A.G., Banat I.M., Mone N.S., Patil R.H., Cameotra S.S. (2016). Biosurfactant/s from *Lactobacilli* species: Properties, challenges and potential biomedical applications. J. Basic Microbiol..

[3] Karamchandani B.M., Pawar A.A., Pawar S.S., Syed S., Mone N.S., Dalvi S.G., Rahman P.K., Banat I.M., Satpute S.K. (2022). Biosurfactants’ multifarious functional potential for sustainable agricultural practices. Front. Bioeng. Biotechnol..

[4] Marchant R., Banat I.M. (2012). Microbial biosurfactants: Challenges and opportunities for future exploitation. Trends Biotechnol..

[5] De Almeida D.G., Soares Da Silva R.D.C.F., Luna J.M., Rufino R.D., Santos V.A., Banat I.M., Sarubbo L.A. (2016). Biosurfactants: Promising molecules for petroleum biotechnology advances. Front. Microbiol..

[6] Campos J.M., Montenegro Stamford T.L., Sarubbo L.A., de Luna J.M., Rufino R.D., Banat I.M. (2013). Microbial biosurfactants as additives for food industries. Biotechnol. Prog..

[7] Campos J.M., Banat I.M., Sarubbo L.A. (2019). Natural and Microbial Biosurfactants’ Use in the Food Industry. Microbial Biosurfactants and Their Environmental and Industrial Applications.

[8] Glynn A., Igra A.M., Sand S., Ilbäck N.G., Hellenäs K.E., Rosén J., Aspenström-Fagerlund B. (2017). Are additive effects of dietary surfactants on intestinal tight junction integrity an overlooked human health risk?—A mixture study on Caco-2 monolayers. Food Chem. Toxicol..

[9] Csáki K.F. (2011). Synthetic surfactant food additives can cause intestinal barrier dysfunction. Med. Hypotheses.

[10] Sharma D. (2021). Biosurfactants: Greener Surface Active Agents for Sustainable Future.

[11] da Silva A.F., Banat I.M., Giachini A.J. (2021). Fungal biosurfactants, from nature to biotechnological product: Bioprospection, production and potential applications. Bioprocess Biosyst. Eng..

[12] Sarubbo L.A., Maria da Gloria C.S., Durval I.J.B., Bezerra K.G.O., Ribeiro B.G., Silva I.A., Twigg M.S., Banat I.M. (2022). Biosurfactants: Production, properties, applications, trends, and general perspectives. Biochem. Eng. J..

[13] Farias C.B.B., Almeida F.C., Silva I.A., Souza T.C., Meira H.M., Rita de Cássia F., Luna J.M., Santos V.A., Converti A., Banat I.M. (2021). Production of green surfactants: Market prospects. Electron. J. Biotechnol..

[14] Sharma D. (2016). Applications of biosurfactants in food. Biosurfactants Food.

[15] Van Haesendonck I., Vanzeveren E. (2003). International patent PCT/BE/2003/000186.

[16] Trummler K., Effenberger F., Syldatk C. (2003). An integrated microbial/enzymatic process for production of rhamnolipids and L-(+)-rhamnose from rapeseed oil with *Pseudomonas* sp. DSM 2874. Eur. J. Lipid Sci. Technol..

[17] Kiran G.S., Priyadharsini S., Sajayan A., Priyadharsini G.B., Poulose N., Selvin J. (2017). Production of lipopeptide biosurfactant by a marine *Nesterenkonia* sp. and its application in food industry. Front. Microbiol..

[18] Stackebrandt E., Koch C., Gvozdiak O., Schumann P. (1995). Taxonomic Dissection of the Genus *Micrococcus*: *Kocuria* gen. nov., *Nesterenkonia* gen. nov., *Kytococcus* gen. nov., *Dermacoccus* gen. nov., and *Micrococcus* Cohn 1872 gen. emend. Int. J. Syst. Evolution. Microbiol..

[19] Kuiper I., Lagendijk E.L., Pickford R., Derrick J.P., Lamers G.E., Thomas-Oates J.E., Lugtenberg B.J.J., Bloemberg Bloemberg G.V. (2004). Characterization of two *Pseudomonas putida* lipopeptide biosurfactants, putisolvin I and II, which inhibit biofilm formation and break down existing biofilms. Molec. Microbiol..

[20] Janek T., Łukaszewicz M., Rezanka T., Krasowska A. (2010). Isolation and characterization of two new lipopeptide biosurfactants produced by *Pseudomonas fluorescens* BD5 isolated from water from the Arctic Archipelago of Svalbard. Bioresour. Technol..

[21] Rivardo F., Turner R.J., Allegrone G., Ceri H., Martinotti M.G. (2009). Anti-adhesion activity of two biosurfactants produced by *Bacillus* spp. prevents biofilm formation of human bacterial pathogens. Appl. Microbiol. Biotechnol..

[22] Pradhan A.K., Pradhan N., Mall G., Panda H.T., Sukla L.B., Panda P.K., Mishra B.K. (2013). Application of lipopeptide biosurfactant isolated from a halophile: *Bacillus tequilensis* CH for inhibition of biofilm. Appl. Biochem. Biotechnol..

[23] Luna J., Rufino R., Campos G., Sarubbo L. (2012). Properties of the biosurfactant produced by *Candida sphaerica* cultivated in low-cost substrates. Chem. Eng..

[24] Dusane D.H., Dam S., Nancharaiah Y.V., Kumar A.R., Venugopalan V.P., Zinjarde S.S. (2012). Disruption of *Yarrowia lipolytica* biofilms by rhamnolipid biosurfactant. Aqua. Biosy..

[25] Rufino R.D., Luna J.M., Sarubbo L.A., Rodrigues L.R.M., Teixeira J.A.C., Campos-Takaki G.M. (2011). Antimicrobial and anti-adhesive potential of a biosurfactant Rufisan produced by *Candida lipolytica* UCP 0988. Coll. Surf. B Biointerfaces.

[26] Dusane D.H., Pawar V.S., Nancharaiah Y.V., Venugopalan V.P., Kumar A.R., Zinjarde S.S. (2011). Anti-biofilm potential of a glycolipid surfactant produced by a tropical marine strain of *Serratia marcescens*. Biofouling.

[27] Benincasa M., Abalos A., Oliveira I., Manresa A. (2004). Chemical structure, surface properties and biological activities of the biosurfactant produced by *Pseudomonas aeruginosa* LBI from soapstock. Antonie Van Leeuwenhoek.

[28] Kim B.S., Lee J.Y., Hwang B.K. (2000). In vivo control and in vitro antifungal activity of rhamnolipid B, a glycolipid antibiotic, against *Phytophthora capsici* and *Colletotrichum orbiculare*. Pest Manag. Sci. Former. Pestic. Sci..

[29] Haba E., Pinazo A., Jauregui O., Espuny M.J., Infante M.R., Manresa A. (2003). Physicochemical characterization and antimicrobial properties of rhamnolipids produced by *Pseudomonas aeruginosa* 47T2 NCBIM 40044. Biotechnol. Bioeng..

[30] Varnier A.L., Sanchez L., Vatsa P., Boudesocque L., Garcia-Brugger A., Rabenoelina F., Sorokin A., Renault J.H., Kauffmann S., Pugin A. (2009). Bacterial rhamnolipids are novel MAMPs conferring resistance to *Botrytis cinerea* in grapevine. Plant Cell Environ..

[31] Nitschke M., Costa S.G., Contiero J. (2010). Structure and applications of a rhamnolipid surfactant produced in soybean oil waste. Appl. Biochem. Biotechnol..

[32] Sotirova A., Spasova D., Vasileva-Tonkova E., Galabova D. (2009). Effects of rhamnolipid-biosurfactant on cell surface of *Pseudomonas aeruginosa*. Microbiol. Res..

[33] Chander C.S., Lohitnath T., Kumar D.M., Kalaichelvan P.T. (2012). Production and characterization of biosurfactant from *Bacillus subtilis* MTCC441 and its evaluation to use as bioemulsifier for food bio-preservative. Adv. Appl. Sci. Res..

[34] Campos J.M., Stamford T.L., Rufino R.D., Luna J.M., Stamford T.C.M., Sarubbo L.A. (2015). Formulation of mayonnaise with the addition of a bioemulsifier isolated from *Candida utilis*. Toxicol. Rep..

[35] Zouari R., Besbes S., Ellouze-Chaabouni S., Ghribi-Aydi D. (2016). Cookies from composite wheat–sesame peels flours: Dough quality and effect of *Bacillus subtilis* SPB1 biosurfactant addition. Food Chem..

[36] Sharma R., Singh J., Verma N. (2018). Production, characterization and environmental applications of biosurfactants from *Bacillus amyloliquefaciens* and *Bacillus subtilis*. Biocat. Agricul. Biotechnol..

[37] Zouari R., Ben Abdallah-Kolsi R., Hamden K., Feki E.A., Chaabouni K., Makni-Ayadi F., Sallemi F., Ellouze-Chaabouni S., Ghribi-Aydi D. (2015). Assessment of the antidiabetic and antilipidemic properties of *Bacillus subtilis* SPB1 biosurfactant in alloxan-induced diabetic rats. Pept. Sci..

[38] Mnif I., Besbes S., Ellouze R., Ellouze-Chaabouni S., Ghribi D. (2012). Improvement of bread quality and bread shelf-life by *Bacillus subtilis* biosurfactant addition. Food Sci. Biotechnol..

[39] Silva I.A., Veras B.O., Ribeiro B.G., Aguiar J.S., Guerra J.M.C., Luna J.M., Sarubbo L.A. (2020). Production of cupcake-like dessert containing microbial biosurfactant as an emulsifier. Peer J..

[40] Vasudevan S., Prabhune A.A. (2008). Photophysical studies on curcumin-sophorolipid nanostructures: Applications in quorum quenching and imaging. R. Soc. Open Sci..

[41] Konkol D., Szmigiel I., Domżał-Kędzia M., Kułażyński M., Krasowska A., Opaliński S., Korczyńskia M., Łukaszewicz M. (2019). Biotransformation of rapeseed meal leading to production of polymers, biosurfactants, and fodder. Bioorganic Chem..

[42] Song B., Zhu W., Song R., Yan F., Wang Y. (2019). Exopolysaccharide from *Bacillus vallismortis* WF4 as an emulsifier for antifungal and antipruritic peppermint oil emulsion. Int. J. Biol. Macromol..

[43] Vidhyalakshmi R., Nachiyar C.V., Kumar G.N., Sunkar S., Badsha I. (2018). Production, characterization and emulsifying property of exopolysaccharide produced by marine isolate of *Pseudomonas fluorescens*. Biocatal. Agri. Bbiotechnol..

[44] Campos J.M., Stamford T.L., Sarubbo L.A. (2014). Production of a bioemulsifier with potential application in the food industry. Appl. Biochem. Biotechnol..

[45] Nitschke M., Costa S.G., Contiero J. (2005). Rhamnolipid surfactants: An update on the general aspects of these remarkable biomolecules. Biotechnol. Prog..

[46] Nitschke M., Costa S.G.V.A.O. (2007). Biosurfactants in food industry. Trends Food Sci. Technol..

[47] Lima Á.S., Alegre R.M. (2009). Evaluation of emulsifier stability of biosurfactant produced by *Saccharomyces lipolytica* CCT-0913. Braz. Arch. Biol. Technol..

[48] Ribeiro B.G., Dos Santos M.M., Pinto M.I., Meira H.M., Durval I.B., Guerra J.M. (2019). Production and optimization of the extraction conditions of a biosurfactant of *Candida utilis* UFPEDA1009 with potential of application in the food industry. Chem. Eng. Trans..

[49] Bai L., McClements D.J. (2016). Formation and stabilization of nanoemulsions using biosurfactants: Rhamnolipids. J. Colloid Interface Sci..

[50] Ely C.M. (1951). Chick-growth stimulation produced by surfactants. Science.

[51] Kim C.H., Kim J.N., Ha J.K., Yun S.G., Lee S.S. (2004). Effects of dietary addition of surfactant Tween 80 on ruminal fermentation and nutrient digestibility of Hanwoo steers. Asian-Australas. J. Anim. Sci..

[52] Gudiña E.J., Rangarajan V., Sen R., Rodrigues L.R. (2013). Potential therapeutic applications of biosurfactants. Trends Pharmacol. Sci..

[53] Goodarzi F., Zendehboudi S. (2019). A comprehensive review on emulsions and emulsion stability in chemical and energy industries. Can. J. Chem. Eng..

[54] Sagalowicz L., Leser M.E. (2010). Delivery systems for liquid food products. Curr. Opin. Colloid Interface Sci..

[55] Farheen V., Saha S.B., Pyne S., Chowdhury B.R. (2016). Production of nanobiosurfactant from *Pseudomonas aeruginosa* and it’s application in bakery industry. Int. J. Adv. Res. Biol. Eng. Sci. Technol..

[56] Khoshdast H., Abbasi H., Sam A., Noghabi K.A. (2012). Frothability and surface behavior of a rhamnolipid biosurfactant produced by *Pseudomonas aeruginosa* MA01. Biochem. Eng. J..

[57] Vaughn S.F., Behle R.W., Skory C.D., Kurtzman C.P., Price N.P.J. (2004). Utilization of sophorolipids as biosurfactants for postemergence herbicides. Crop. Prot..

[58] Randu M., Sylvie H.E.R.Y., Ravier P., Deprey S. (2021). Concentrate comprising a MEL and a polyethylene glycol fatty acid ester having an HLB value greater than or equal to 12. U.S. Patent.

[59] Sekhar K.P., Adicherla H., Nayak R.R. (2018). Impact of glycolipid hydrophobic chain length and headgroup size on self-assembly and hydrophobic guest release. Langmuir.

[60] De Zoysa G.H., Glossop H.D., Sarojini V. (2018). Unexplored antifungal activity of linear battacin lipopeptides against planktonic and mature biofilms of *C. albicans*. European J. Med. Chem..

[61] Braun A.C., Ilko D., Merget B., Gieseler H., Germershaus O., Holzgrabe U., Meinel L. (2015). Predicting critical micelle concentration and micelle molecular weight of polysorbate 80 using compendial methods. Eur. J. Pharm. Biopharm..

[62] Gaur V.K., Regar R.K., Dhiman N., Gautam K., Srivastava J.K., Patnaik S., Manickam N. (2019). Biosynthesis and characterization of sophorolipid biosurfactant by *Candida* spp.: Application as food emulsifier and antibacterial agent. Bioresour. Technol..

[63] Bjerk T.R., Severino P., Jain S., Marques C., Silva A.M., Pashirova T., Souto E.B. (2021). Biosurfactants: Properties and applications in drug delivery, biotechnology and ecotoxicology. Bioengineering.

[64] Durval I.J.B., da Silva I.A., Sarubbo L.A. (2021). Application of microbial biosurfactants in the food industry. Microbial Biosurfactants.

[65] Ozdener M.H., Ashby R.D., Jyotaki M., Elkaddi N., Spielman A.I., Bachmanov A.A., Solaiman D.K. (2019). Sophorolipid biosurfactants activate taste receptor type 1 member 3-mediated taste responses and block responses to bitter taste in vitro and in vivo. J. Surfact. Deter..

[66] Magalhães L., Nitschke M. (2013). Antimicrobial activity of rhamnolipids against *Listeria monocytogenes* and their synergistic interaction with nisin. Food Control.

[67] Zhang J., Bilal M., Liu S., Zhang J., Lu H., Luo H., Zhao Y. (2020). Isolation, identification and antimicrobial evaluation of bactericides secreting *Bacillus subtilis* natto as a biocontrol agent. Processes.

[68] Partal P., Guerrero A., Berjano M., Gallegos C. (1999). Transient flow of o/w sucrose palmitate emulsions. J. Food Eng..

[69] Kralova I., Sjöblom J. (2009). Surfactants used in food industry: A review. J. Dispers. Sci. Technol..

[70] Twigg M.S., Tripathi L., Zompra A., Salek K., Irorere V.U., Gutierrez T., Spyroulias G.A., Marchant R., Banat I.M. (2018). Identification and characterization of short chain rhamnolipid production in a previously uninvestigated, non-pathogenic marine *Pseudomonas*. Appl. Microbiol. Biotechnol..

[71] Tripathi L., Twigg M.S., Zompra A., Salek K., Irorere V.U., Gutierrez T., Spyroulias G.A., Marchant R., Banat I.M. (2019). Biosynthesis of rhamnolipid by a *Marinobacter* species expands the paradigm of biosurfactant synthesis to a new genus of the marine microflora. Microb. Cell Fact..

[72] Jeniel Biotech Inc Home Page. https://www.jeneilbiotech.com.

[73] Marchant R., Banat I.M. (2012). Biosurfactants: A sustainable replacement for chemical surfactants?. Biotechnol. Lett..

[74] Sharma P., Madhyastha H., Madhyastha R., Nakajima Y., Maruyama M., Verma K.S., Verma S., Prasad J., Kothari S.L., Gour V.S. (2019). An appraisal of cuticular wax of *Calotropis procera* (Ait.) R. Br.: Extraction, chemical composition, biosafety and application. J. Hazard. Mater..

[75] Sharma V., Singh D., Manzoor M., Banpurkar A.G., Satpute S.K., Sharma D. (2022). Characterization and cytotoxicity assessment of biosurfactant derived from *Lactobacillus pentosus* NCIM 2912. Braz. J. Microbiol..

[76] Rodríguez-López L., López-Prieto A., Lopez-Álvarez M., Pérez-Davila S., Serra J., González P., Moldes A.B. (2020). Characterization and cytotoxic effect of biosurfactants obtained from different sources. ACS Omega.

[77] (2022). Biological evaluation of medical devices—Part 5: Tests for in Vitro Cytotoxicity.

[78] Voulgaridou G.-P., Mantso T., Anestopoulos I., Klavaris A., Katzastra C., Kiousi D.-E., Mantela M., Galanis A., Gardikis K., Banat I.M. (2021). toxicity profiling of biosurfactants produced by novel marine bacterial strains. Int. J. Mol. Sci..

[79] Adu S.A., Twigg M.S., Naughton N.J., Marchant R., Banat I.M. (2023). Characterization of cytotoxicity and immunomodulatory effects of glycolipid biosurfactants on human keratinocytes. Appl. Microbiol. Biotechnol..

[80] Food and Agriculture Organization (2020). SAVE FOOD: Global Initiative on Food Loss and Waste Reduction|Key Facts on Food Loss and Waste You should Know!. http://www.fao.org/save-food/resources/keyfindings/en/.

[81] Cofelice M., Cuomo F., Chiralt A. (2019). Alginate films encapsulating lemongrass essential oil as affected by spray calcium application. Coll. Interf..

[82] Meira S.M.M., Zehetmeyer G., Werner J.O., Brandelli A. (2017). A novel active packaging material based on starch-halloysite nanocomposites incorporating antimicrobial peptides. Food Hydrocol..

[83] Zainal Abidin M., Kourmentza C., Karatzas A.K., Niranjan K. (2019). Enzymatic hydrolysis of thermally pre-treated chitin and antimicrobial activity of N, N’-diacetylchitobiose. J. Chem. Technol. Biotechnol..

[84] Meena K.R., Sharma A., Kanwar S.S. (2019). Antitumoral and antimicrobial activity of surfactin extracted from Bacillus subtilis KLP2015. Int. J. Pept. Res. Ther..

[85] Kourmentza K., Gromada X., Michael N., Degraeve C., Vanier G., Ravallec R., Jauregi P. (2021). Antimicrobial activity of lipopeptide biosurfactants against foodborne pathogen and food spoilage microorganisms and their cytotoxicity. Front. Microbiol..

[86] Thimon L., Peypoux F., Wallach J., Michel G. (1995). Effect of the lipopeptide antibiotic, iturin A, on morphology and membrane ultrastructure of yeast cells. FEMS Microbiol. Lett..

[87] Sánchez M., Teruel J.A., Espuny M.J., Marqués A., Aranda F.J., Manresa Á., Ortiz A. (2006). Modulation of the physical properties of dielaidoylphosphatidylethanolamine membranes by a dirhamnolipid biosurfactant produced by *Pseudomonas aeruginosa*. Chem. Phys. Lipids.

[88] Sotirova A.V., Spasova D.I., Galabova D.N., Karpenko E., Shulga A. (2008). Rhamnolipid–biosurfactant permeabilizing effects on gram-positive and gram-negative bacterial strains. Curr. Microbiol..

[89] Manzoor M., Singh D., Aseri G.K., Sohal J.S., Vij S., Sharma D. (2021). Role of lacto-fermentation in reduction of antinutrients in plant-based foods. J. Appl. Biol. Biotechnol..

